# 

**Published:** 1892-02

**Authors:** 


### SPECIAL.

We very much desire the June, August, and September, 1888, numbers of our JOURNAL OF HEALTH, to complete a set of that year. Will some good friend send us either one or all of said numbers?

### LITERARY.

Vick's Floral Guide, 1892, 77 pp., 4to.—The handsomest yet; indeed it would be difficult to conceive a more complete and tasteful guide of its class. The catalogue embraces some five hundred varieties of selected flowers, and the illustrated text is so clear that, in ordering seeds, no one can go amiss. Then, too, the notes on the culture of the different specimen plants are worth the whole cost of the book. The horticultural department is equally commendable. Send orders to James Vicks Sons, Rochester, N. Y., and get what you call for, as you will, but don't expect so much of Rose Hill.

The Dentist Himself—A new Monthly.—By J. A. Kimball. We have just a word to say of Dr. Kimball and his first born. The publisher, aside from being a practical dentist of many years experience, is an admirable writer not unknown in the field of letters, "knows of his own knowledge," the wants of his fellow operatives in a profession where the best means and highest skill are always in requisition, and none other that the Dentist Himself can fill the bill. The only fault we find with this newly fledged candidate for dental favor is that it is worth five times the money asked for it.

Medical Hemorrhage Surgically Treated.—By Professor Robert H. N. Dawbarn, M.D.

This is a very instructive paper abounding with interesting details of the author's practice in support of his claims. We commend his frankness in saying "The very fact that the list of remedies at our command, and recommended by standard authorities as of some use, is so long a one of itself, goes to show their inefficiency."

In this we quite agree.

Stricture of the Rectum; a study of 138 cases, illustrated. By Professor Charles B. Kelsey, M.D.

This is a most valuable treatise for practitioners and students, and reflects great credit upon its scientific author.

A B C of Swedish Educational Gymnastics.—By Professor Harting Nissen. 77 illustrations, 107 pp. F. A. Davis, Publisher, Philadelphia. Price, 75 cents, flexible cloth covers.

The above is a "Practical Hand Book for School Teachers," but every movement is so clearly defined and illustrated, that the book will serve equally well for families and individuals. We do not hesitate to recommend it as a perfect manual of exercise, suited to develop every part of the human frame to its utmost capacity without the use of cumbersome apparatus.

We are under obligations for an advance copy of the THIRD ANNUAL REPORT on the STATISTICS of RAILWAYS in the UNITED STATES, for the year ending June 1890, to the INTERSTATE COMMERCE COMMISSION.

This report is of great commercial value as showing the route, extent and earnings of the several roads composing the vast railroad system of our country.

NEW YORK has one REFORMATORY INSTITUTION of which she may be justly proud, located at Elmira, whose sixteenth YEAR BOOK presents a remarkably encouraging showing. It is encouraging to note that minor offences are condoned rather than punished at this humane retreat, and the offenders fairly educated and taught useful and self-supporting trades.

THE JOURNALIST—HOLIDAY SPECIAL for DECEMBER, 1891, is a model of typographical neatness, electro-photography, and general excellence. May it "live long and prosper."

The several authors of the following treatises will accept our thanks:

The Forms and Treatment of Club-foot, etc., etc.—By H. Augustus Wilson, M.D., Professor of Surgery, in the College of Graduates, and Jefferson Medical College of Philadelphia, etc.

AN INTERESTING REPORT of a case of Spina Bifida.—By Professor H. Augustus Wilson, Medical College, Philadelphia.

A PACK OF FOOLS.—By Dr. Henry S. Chase, St. Louis. Illustrated, showing by contrast, a *natural woman* and an *artificial lady*—with many suggestions of value to the gentle sex.

SHORTHAND AND TYPEWRITING.—By Dugald McKillop. Illustrated, 125 pp. Fowler & Wells Co., 775 Broadway, New York City. Price, 40 cents.

This is an admirable treatise on the two arts which have become so necessary to the court room, counting house and business in its various forms. For students this little wlli be found of value.

